# Vergleich der subjektiven Lebensqualitätsverbesserung von Patienten nach eröffnenden und minimal-invasiven Operationstechniken zur Rekanalisierung von Tränenwegsstenosen in den Jahren 2015 bis 2018

**DOI:** 10.1007/s00347-021-01400-w

**Published:** 2021-05-17

**Authors:** J. Mehlan, F. Ismani, S. Dulz, S. Green, M. S. Spitzer, F. Schüttauf

**Affiliations:** Klinik und Poliklinik für Augenheilkunde, Universitätsklinikum Hamburg-Eppendorf, Martinistr. 52, 20246 Hamburg, Deutschland

**Keywords:** Tränenwegschirurgie, Patientenzufriedenheit, Dakryozystorhinostomie, Tränenwegsendoskopie, Epiphora, Lacrimal duct surgery, Patient satisfaction, Dacryocystorhinostomy, Lacrimal endoscopy, Epiphora

## Abstract

**Hintergrund:**

Die eröffnende und die minimal-invasive Tränenwegschirurgie gehören zu den häufigen Operationsindikationen. Jedoch ist bislang wenig über die jeweilige Beeinflussung der Lebensqualität bekannt.

**Ziel der Arbeit:**

Mit dieser Studie soll die subjektive Beeinflussung der Lebensqualität von Patienten nach eröffnenden und minimal-invasiven Operationstechniken zur Rekanalisierung von Dakryostenosen vergleichend erfasst werden.

**Material und Methoden:**

Aus dem Kollektiv der Patienten, die von 2015 bis 2018 am Universitätsklinikum Hamburg-Eppendorf operiert wurden, nahmen 169 Patienten (111 DCR, 58 Endoskopie) an der Umfrage teil und beantworteten 9 Fragen zur subjektiven Zufriedenheit, welche wir – auch vergleichend – ausgewertet haben.

**Ergebnisse:**

Gefragt nach der postoperativen Zufriedenheit zeigten sich die Patienten nach DCR signifikant zufriedener (*p* = 0,001) als die Patienten, die eine Tränenwegsendoskopie erhielten. Es zeigte sich kein signifikanter Unterschied hinsichtlich der postoperativen Komplikationen (*p* = 0,348). Die Rate an Re-Operationen jedoch war in der Patientengruppe, welche eine Tränenwegsendoskopie erhielten, signifikant höher (Chi-Quadrat-Test, *p* = 0,004).

**Schlussfolgerung:**

Zusammenfassend lässt sich daher sagen, dass die DCR hinsichtlich der Patientenzufriedenheit einer Tränenwegsendoskopie nicht unterlegen ist.

## Abstract (en)

**Background:**

Open and minimally invasive tear duct surgery are among the common surgical indications; however, little is known so far about the respective influences on the quality of life.

**Objective:**

The aim of this study was to compare the subjective influence on the quality of life of patients after open and minimally invasive surgical techniques for recanalization of dacryostenosis.

**Material and methods:**

From the collective of patients who were surgically treated at the University Medical Center Hamburg-Eppendorf from 2015 to 2018, a total of 169 patients (111 dacryocystorhinostomy, DCR, 58 endoscopy) took part in the survey and answered 9 questions about subjective satisfaction, which were evaluated also comparatively.

**Results:**

When asked about postoperative satisfaction, the patients were significantly more satisfied after DCR (*p* = 0.001) than the patients who underwent a lacrimal endoscopy. There was no significant difference in terms of postoperative complications (*p* = 0.348). The rate of reoperations, however, was significantly higher in the patient group who underwent lacrimal endoscopy (χ^2^-test, *p* = 0.004).

**Conclusion:**

In summary it can be said that DCR is not inferior to lacrimal endoscopy in terms of patient satisfaction.

Es existieren in der Tränenwegschirurgie sowohl eröffnende, als auch minimal-invasive Techniken zur Behandlung von Dakryostenosen.

Beide Operationsverfahren können einem unterschiedlichen Indikationsspektrum zugeordnet werden. Während die Tränenwegsendoskopie vorrangig bei präsaccalen oder funktionellen Stenosen zum Einsatz kommt, liegt das Indikationsgebiet der Dacryozystorhinostomie (DCR) v. a. im Bereich der Tränenwegsverschlüsse, v. a. postsaccal.

Die Standardprozedur der eröffnenden Tränenwegsoperation ist die DCR, welche über einen Hautschnitt eine Verbindung von Tränensack zur Nasenhöhle über eine Osteotomie ermöglich. Im Gegensatz dazu benötigt die Tränenwegsendoskopie, welche die minimal-invasive Technik repräsentiert, keinen Hautschnitt. Am Ende beider operativer Prozeduren wird eine bikanalikuläre Intubation für 3 Monate eingelegt.

In vorangegangenen Studien konnte gezeigt werden, dass nach der DCR eine hohe Zufriedenheit der Patienten herrscht [3], aber auch die Erfolgsraten werden mit bis zu 99 % in der Literatur angegeben [4].

Gerade auch dadurch, dass die endoskopischen Techniken sich in den letzten Jahren sehr weiterentwickelt und auch an Einfluss gewonnen haben [2], ist ein Vergleich der subjektiven Lebensqualitätsverbesserung postoperativ sowie v. a. auch der Vergleich zwischen den beiden Prozeduren anzustreben und von hoher klinischer Bedeutung.

Ziel der hier vorgelegten Arbeit ist es daher, die subjektive Beeinflussung der Lebensqualität von Patienten nach eröffnenden und minimal-invasiven Operationstechniken zur Rekanalisierung von Dakryostenosen zu erfassen.

Somit können wesentliche Aussagen zur Beratung und Aufklärung der Patienten gewonnen werden.

## Methodik

Verglichen werden 2 unterschiedliche Operationsansätze (eröffnende vs. minimal-invasive Tränenwegschirurgie) hinsichtlich ihrer subjektiv empfundenen Lebensqualitätsverbesserung postoperativ bzw. auch hinsichtlich der Zufriedenheit mit der Operationsprozedur und dem Operationsergebnis in den Jahren 2015 bis 2018.

Die Indikationsstellung für die Operationen erfolgte im Vorwege gänzlich unabhängig von der vorgelegten Studie. Im Zuge der ambulanten Vorstellung in unseren Sprechstunden erfolgte eine Tränenwegsspülung zu diagnostischen Zwecken. Bei präsaccalen oder funktionellen Stenosen kam vorrangig die Tränenwegsendoskopie zum Einsatz. Die Dakryozystorhinostomie wurde bei kompletten Tränenwegsverschlüssen oder nach Infektionen indiziert.

Alle betroffenen Patienten wurden gebeten, einen Fragenbogen retrospektiv hinsichtlich der präoperativen Symptome, der dadurch resultierenden Einschränkung der Lebensqualität, ihres Erlebens der operativen Prozedur sowie der Veränderung der Lebensqualität postoperativ auszufüllen; 169 Patienten (111 DCR, 58 Endoskopie) nahmen an der Umfrage teil und beantworteten 9 Fragen zur subjektiven Zufriedenheit.

Die Daten der Patienten wurden vor der Erhebung anonymisiert.

In die Erhebung wurden keine Re-Operationen mit eingeschlossen, um eine Vermischung des subjektiven Erlebens zwischen Erstversorgung sowie Rezidiv zu vermeiden.

Die Indikationsstellung erfolgte im Rahmen unserer okuloplastischen Sprechstunden nach Anamnese, klinischer Untersuchung und diagnostischer Tränenwegsspülung. Bei Verschlüssen oder rezidivierenden Entzündungen wurde eine DCR empfohlen. Im Falle von relativen Stenosen v. a. im präsaccalen Bereich oder wenn eine Vermeidung von Hautnarben für den Patienten wichtig war, erfolgte eine Tränenwegsendoskopie – bei Letzteren unter Hinweis auf das ggf. erhöhte Rezidivrisiko.

Alle Prozeduren wurden von 4 erfahrenen Operateuren durchgeführt.

### Dakryozystorhinostomie

Unter Vollnarkose wird nach vorheriger Hautdesinfektion ein Hautschnitt von ca. 2 cm im Bereich der Fossa lacrimalis durchgeführt und stumpf bis auf den Nasenknochen präpariert, das Periost inzidiert und vom Knochen geschoben. Mithilfe einer Knochenstanze oder eines Bohrers wird im Os lacrimale und Processus frontalis ein Knochenfenster eröffnet. Die Nasenschleimhaut und der Tränensack werden türflügelartig eröffnet und anastomosiert. Somit kann die Tränenflüssigkeit so aus dem Tränensack direkt in den mittleren Nasengang abgeleitet werden.

Zum Ende der Operation erfolgte die Intubation mit einem Silikonschlauch. Die Verweildauer der Intubation lag wie bei der Tränenwegsendoskopie bei 3 Monaten.

### Tränenwegsendoskopie

Die Prozeduren erfolgten allesamt in Allgemeinanästhesie. Bei unseren Patienten wurde das PolyShaft®-Endoskop der Firma Bess Medizintechnik, Berlin, eingesetzt.

Zunächst wurden die Tränenpünktchen dilatiert und anschließend das Endoskop eingebracht und unter Spülung bis in den unteren Nasengang vorgeschoben. Für die Beurteilung des Gangsystems wurde das Endoskop kontinuierlich zurückgezogen, und unter Spülung erfolgte die genaue Beurteilung des Gangsystems. Im Falle einer Stenose wurde diese bei unserem Patientenkollektiv mittels Mikrodrilldakryoplastik eröffnet.

### Fragebogen

**Figure 1:**
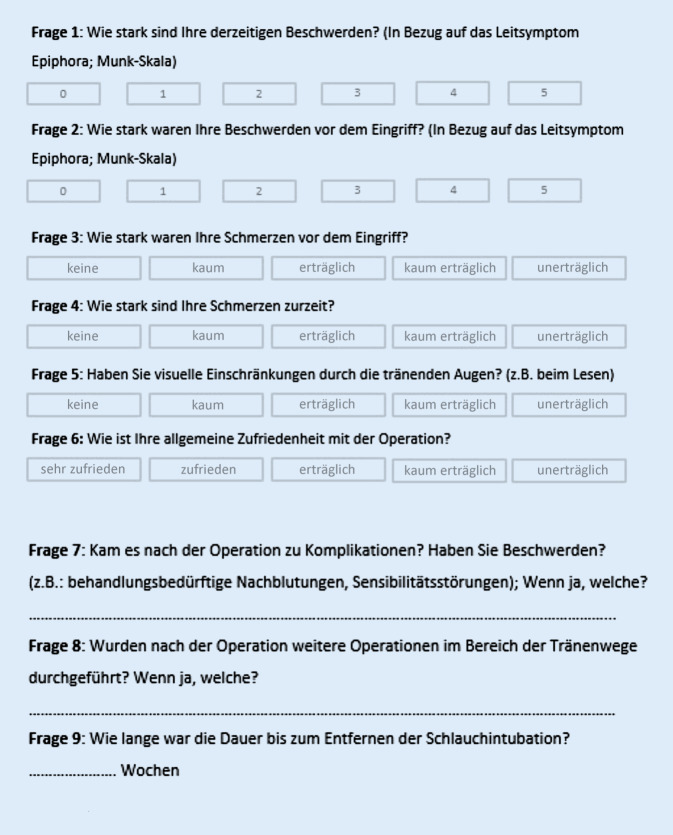

Es wurden alle Patienten anonymisiert und im Zuge der postoperativen Nachbeobachtung gebeten, den Fragebogen, bestehend aus 9 Fragen (Abb. 1), auszufüllen. Für die Antwortmöglichkeit wurde ein Scoring von 1 bis 5 vorgegeben.

Wir haben uns bei der Auswahl des Fragebogens an einem in unserem Haus bereits zuvor bestehenden Fragebogen orientiert. Dieser wurde bereits im Vorwege designed und diente der Evaluation der Patientenzufriedenheit nach tränenwegschirurgischen Eingriffen in den Jahren 2009 bis 2014 und wurde im Rahmen einer Dissertation angewendet. Da sich in den darauffolgenden Jahren nicht nur das Personal in der Abteilung verändert hat, sondern auch beim Bestandspersonal mehr Erfahrung vorlag, interessierte uns, inwiefern eine Veränderung der Zufriedenheit vorlag, auch um unser eigenes Arbeiten zu evaluieren.

Die Fragen, die wir im Rahmen dieser Arbeit den Patienten stellten, entstammten einer Kombination der klinisch anerkannten Munk-Skala (Fragen 1 und 2) [5] und auch in Anlehnung an den SF-12 speziell präzisierten Fragen, um die Symptome dieses Krankheitsbildes abfragen zu können. So wurden unsere Fragen 3 und 4 gemäß der Frage 8 aus dem SF-12 abgewandelt, und beispielsweise Frage 6 ist eine Modifikation der Fragen 9 bis 11 aus dem SF-12 [7].

Mit den Fragen 7 bis 9 unseres Fragebogens wollten wir Fakten wie Komplikationen, Reoperationen und Dauer der Intubation erfragen.

### Statistische Auswertung

Die aus dem Fragebogen resultierenden ordinalskalierten Merkmale wurden mittels Man-Whitney-Test zwischen den Gruppen verglichen. Die Veränderung von präoperativen zu postoperativen Werten wurde mittels Wilcoxon-Vorzeichen-Test innerhalb der Gruppen geprüft. Die Interaktion von Veränderung und Gruppenzugehörigkeit wurde mittels nichtparametrischer Varianzanalyse analysiert. Die beschreibende Statistik wurde in Form von Medianen und Quartile berichtet. Die Häufigkeiten von Komplikationen und Nachbehandlungen wurden mittels Chi-Quadrat-Test zwischen den Gruppen verglichen.

## Ergebnisse

Es konnten die Antworten von 169 Patienten (111 DCR, 58 Endoskopie) ausgewertet werden (Tab. 1).

**Table 1:** 

	DCR	Endoskopie	*p*-Value
*n*	111	58	–
*Beschwerden*			
Vor Operation	5,00 [4,00, 5,00]	5,00 [4,00, 5,00]	0,348
Nach Operation	0,00 [0,00, 2,00]	0,50 [0,00, 3,00]	0,167
*Schmerzen*			
Vor Operation	0,00 [0,00, 2,00]	0,00 [0,00, 0,00]	0,005
Nach Operation	000 [0,00, 0,00]	0,00 [0,00, 0,00]	0,742
*Visuelle Einschränkungen*			
Vor Operation	2,00 [0,00, 3,00]	2,00 [0,00, 3,00]	0,822
Nach Operation	0,00 [0,00, 0,00]	0,00 [0,00, 2,00]	0,145
*Zufriedenheit*	0,00 [0,00, 1,00]	1,50 [0,00, 4,00]	0,001
*Komplikationen*	12 (10,8)	3 (5,2)	0,267
*Nachbehandlung*	8 (7,2)	14 (24,1)	0,003
*Tag bis Schlauchintubation*	86,00 [65,00, 101,00]	86,00 [62,00, 105,25]	0,0905

Da unterschiedliche Gruppenstärken vorlagen, haben wir die Effektstärke nach Cohen zusätzlich bestimmt [1]. Die Stichprobengröße (58 Endoskopie; 111 DCR) war ausreichend, um einen mittleren Effekt beim Unterschied der ordinalskalierten Variablen (Effektstärke d nach Cohen* =−0,5) mit Teststärke von 80 % und Alpha-Fehler von 0,05 statistisch belegen zu können.

Möglich waren Angaben entsprechend eines Scoring-Systems von 0 (keine) bis 5 (maximal) für die Beschwerden, Schmerzen und visuelle Einschränkungen.

Bei der Frage nach der Zufriedenheit steht die 0 für sehr zufrieden und die 5 für sehr unzufrieden.

Zu beachten ist, dass in der Tab. 1 Mediane und Quartile als richtige Maße für zentrale Tendenz und Streuung aufgrund der ordinalskalierten Variablen eingesetzt sind.

Es zeigten sich postoperativ bei 12 Patienten mit DCR und 3 Patienten mit Tränenwegsendoskopie Komplikationen. Diese Komplikationen umfassen Schlauchdislokation, Sensibilitätsstörung im Bereich der Operationsnarbe (1 Patient), endonasaler Abszess postoperativ (*n* = 1) sowie auch erhöhte Schlauchmobilität postoperativ.

Der Unterschied zwischen den beiden Gruppen war jedoch statistisch nicht signifikant.

Bis zur Entfernung der Schlauchintubation vergingen im Durchschnitt 86 Tage bei den Patienten, die eine DCR erhielten, und 89 Tage bei dem Patientenkollektiv, das eine Tränenwegsendoskopie erhielt (Tab. 1).

Der Unterschied ist statistisch nicht signifikant (*p* = 0,905).

Einen signifikanten Unterschied (*p* = 0,001) konnten wir jedoch bei der postoperativen Zufriedenheit nachweisen. Bei der DCR zeigten sich die Patienten sehr zufrieden (Median 0), und nach der Tränenwegsendoskopie ergab sich ein Median von 1,5.

**Figure 2:**
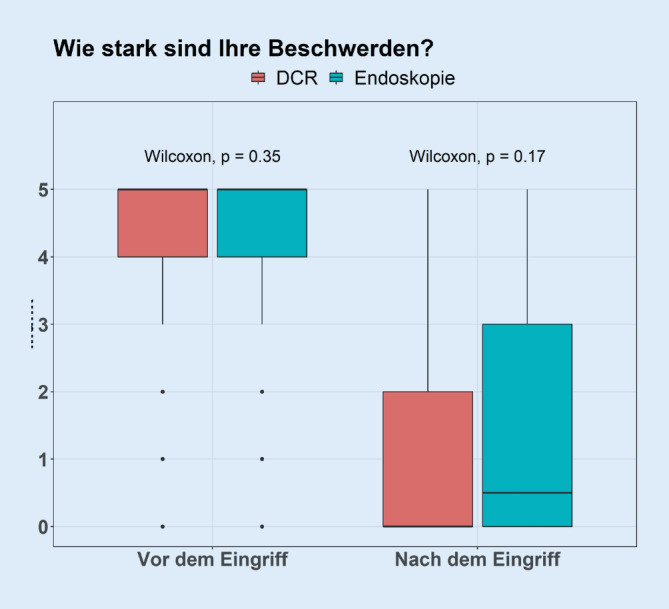

In der Abb. 2 zeigt sich, dass die Veränderungen von prä- zu postoperativ jeweils hochsignifikant sind (Wilcoxon-Vorzeichen-Test *p* < 0,001). Dies betrifft den Unterschied sowohl innerhalb der Gruppen als auch global. Die Interaktion ist nicht signifikant. Das bedeutet, man kann nicht davon ausgehen, dass die Veränderung in der Gruppe der DCR-Patienten stärker war als in der Gruppe der Endoskopiepatienten.

**Figure 3:**
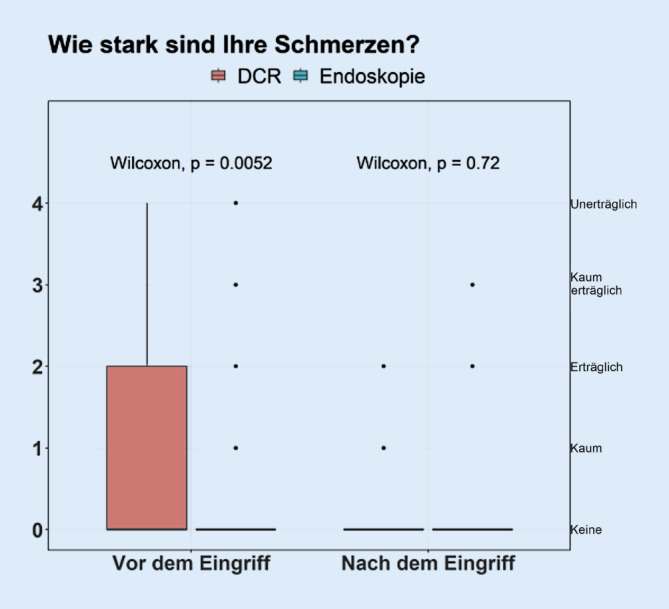

In der Abb. 3 werden die Schmerzen prä- sowie auch postoperativ vergleichend dargestellt. Die Veränderung von prä- zu postoperativ ist signifikant im Patientenkollektiv, welches eine DCR erhielt (*p* < 0,001). Die Werte in Endoskopie bleiben unverändert, *p* = 0,178. Diese Interaktion ist signifikant (*p* = 0,004).

**Figure 4:**
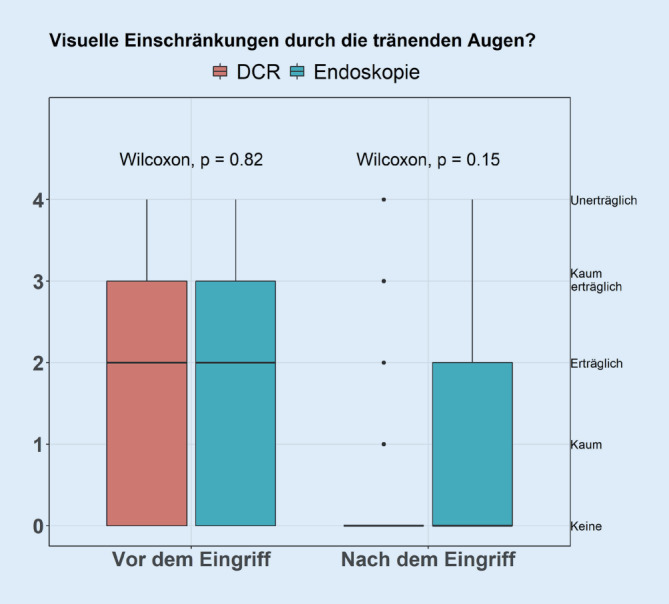

Die Patienten wurden zudem nach visuellen Einschränkungen durch die Epiphora gefragt.

Es zeigte sich eine deutliche Reduktion der empfundenen Einschränkung bei beiden Patientenkollektiven. Die Änderung prä- zu postoperativ bezüglich der empfundenen visuellen Einschränkung war statistisch signifikant in beiden Gruppen (Wilcoxon-Vorzeichen-Test *p* < 0,001 in beiden Gruppen). Die Interaktion zwischen Gruppe und Zeit ist nicht signifikant. Das bedeutet, man kann nicht davon ausgehen, dass die Veränderung in der Gruppe der DCR stärker war als in der Gruppe der Endoskopien.

**Figure 5:**
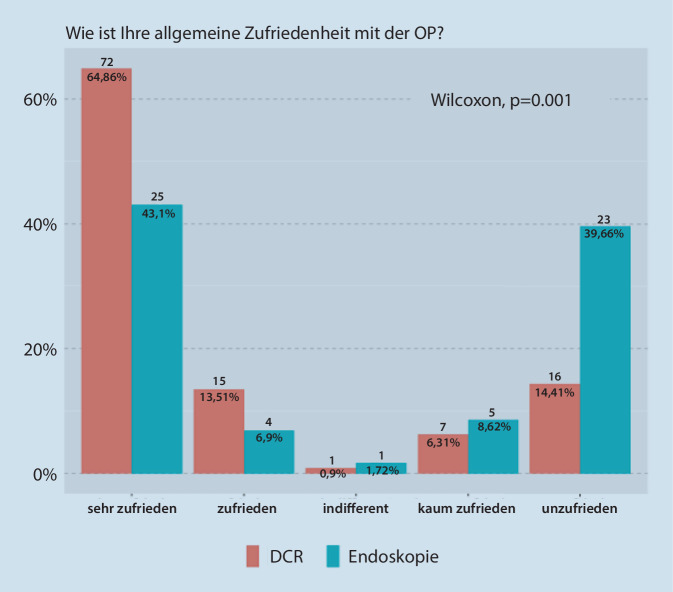

In der Abb. 5 ist die Zufriedenheit der Pateinten vergleichend zwischen den beiden Patientenkollektiven dargestellt worden. Die Patienten, die eine DCR erhielten, zeigten sich signifikant zufriedener (*p* = 0,001), als diejenigen, die eine Tränenwegsendoskopie erhielten.

Wir möchten hierbei darauf hinweisen, dass in der Tab. 1 Mediane und Quartile als richtige Maße für zentrale Tendenz und Streuung aufgrund der ordinalskalierten Variablen eingesetzt sind. Im Fall der Patientenzufriedenheit (0,00 [0,00, 1,00]) gibt der Median an, dass mindestens 50 % der Fälle sehr zufrieden waren (weil 0 für „Sehr zufrieden“ steht). Der erste Wert in den Klammern ist auch 0, also mindestens 25 % aller Fälle sehr zufrieden waren. Der zweite Wert in den Klammern ist 1, also 75 % der Fälle waren mindestens zufrieden (weil 1 für „zufrieden“ steht).

**Figure 6:**
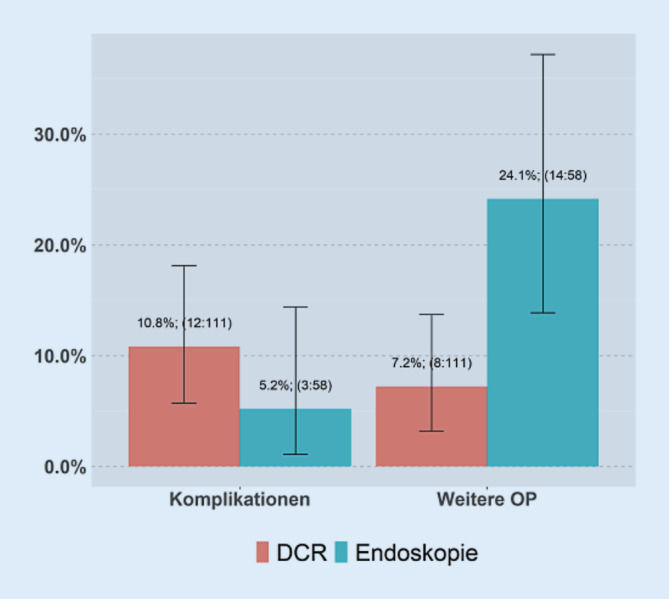

In der Abb. 6 sind die von den Patienten im Zuge dieser Befragung angegebenen Komplikationen aufgetragen. Nach der DCR gaben 10,8 % der Patienten Komplikationen wie beispielsweise Schlauchdislokationen, erhöhte Mobilität der Intubation, einen postoperativen Abszess sowie 1 Patient gab Sensibilitätsstörungen im Bereich der Operationsnarbe an. Nach der Endoskopie waren es lediglich 5,2 % der Patienten die Komplikationen wie Schlauchdislokationen oder erhöhte Mobilität der Intubation angaben. Bei der Betrachtung der Komplikationen ist der Unterschied aber nicht statistisch signifikant (*p* = 0,348).

Die Rate an Re-Operationen war in der Patientengruppe, welche eine Tränenwegsendoskopie erhielten, signifikant höher (Chi-Quadrat-Test, *p* = 0,004). Das zeigt sich auch an den nicht überschneidenden Konfidenzintervallen.

## Diskussion

In der vorgelegten Analyse sollen die subjektive Einschränkung der Lebensqualität sowohl durch die Tränenwegsstenosen als auch aus der operativen Versorgung, offen chirurgisch oder endoskopisch, im Vergleich dargestellt werden. Grundsätzlich muss bemerkt werden, dass für beide Operationsverfahren unterschiedliche Indikationen bestehen und dementsprechend auch 2 verschiedene Patientenkollektive miteinander verglichen werden.

Die Tränenwegsendoskopie kommt vorrangig bei präsaccalen oder funktionellen Stenosen zum Einsatz. Die DCR ist bei Verschlüssen oder rezidivierenden Entzündungen indiziert.

Die beiden Gruppen, konnten rein statistisch betrachtet, trotz unterschiedlicher Gruppengröße, miteinander verglichen werden, da gemäß der Effektstärke nach Cohen die Stichprobengröße ausreichend ist, um einen mittleren Effekt beim Unterschied der ordinalskalierten Variablen statistisch belegen zu können.

Interessanterweise zeigte sich postoperativ kein signifikanter Unterschied hinsichtlich der Schmerzen zwischen DCR und Tränenwegsendoskopie.

Signifikante Unterschiede konnten wir jedoch bei der Frage nach der Zufriedenheit mit der Operation nachweisen.

Die Patienten, die eine DCR erhielten, zeigten sich signifikant zufriedener als diejenigen, welche eine Tränenwegsendoskopie erhielten.

Trotz des Hautschnitts und einer entsprechend oft diskutierten kosmetischen Beeinträchtigung waren die Patienten zufriedener. Es zeigten sich postoperativ zwar weniger Komplikationen bei den Endoskopiepatienten, dennoch geringfügig mehr postoperative Beschwerden.

Diese Beschwerden lassen sich ggf. auch mit dem Patientenkollektiv eher korrelieren als denn mit der Prozedur an sich.

Im Jahr 2015 führten Stemplewitz et al. in unserem Haus eine Evaluation der Ergebnisse nach minimal-invasiver Tränenwegschirurgie bei funktionellen Tränenwegsstenosen durch. Es wurden hierfür die postoperativen Ergebnisse von 118 Patienten, die im Jahr 2010 mit minimal-invasiver Technik versorgt wurden, evaluiert [6].

Dieses Kollektiv wurde aufgrund des Zeitraums der damaligen Untersuchungen nicht in die vorgelegte Studie eingeschlossen. Die Erhebung inkludierte auch ein Telefoninterview mit der Frage nach der Veränderung der Beschwerden durch die minimal-invasive Prozedur.

Es gaben 90 von 118 Patienten (76 %) im Zuge dieses Telefoninterviews eine Verbesserung der präoperativen Beschwerden an; 16 % der Patienten gaben unveränderte Beschwerden an, und 8 % empfanden eine Verschlechterung.

Vergleichbar haben wir in unserer Analyse nach den präoperativen Beschwerden und der Entwicklung durch die Operation gefragt. Es zeigte sich ebenso wie in der oben erwähnten nicht nur eine signifikante Verbesserung der empfundenen Beschwerden nach der Endoskopie, sondern auch nach der DCR.

Präoperativ bestand bei allen Patienten eine mittlere Beschwerdestärke von „5“ und nach der DCR gemäß unserer Skala von „0“ und nach der Tränenwegsendoskopie von „0,5“.

Auffällig ist, dass bei den Patienten, die eine Tränenwegsendoskopie erhielten, postoperativ mehr Beschwerden angaben, als die Patienten, die eine DCR erhielten. Statistisch war dieser Unterschied jedoch nicht signifikant.

Anders gestaltet sich jedoch die Angabe von Komplikationen, die die Patienten machten.

Von den Patienten, die einer Tränenwegsendoskopie unterzogen wurden, gaben lediglich 5,2 % an, postoperativ Komplikationen gehabt zu haben, wohingegen 10,8 % nach DCR angaben, Komplikationen gehabt zu haben. Auch dieser Unterschied war statistisch nicht signifikant.

Wir planen allerdings weitere Analysen im Bereich der Versorgungsforschung, sodass solcherlei Auffälligkeiten hoffentlich künftig mit dem individuellen Verlauf korreliert werden können.

Die Zufriedenheit des Patientenkollektivs, das eine DCR erhielt, war signifikant höher (*p* = 0,001; vgl. Tab. 1 und Abb. 5) als die Zufriedenheit bei denjenigen, die eine Tränenwegsendoskopie bekamen.

Diesen Unterschied zu klären fällt zunächst schwer. Es zeigte sich kein signifikanter Unterschied hinsichtlich der Schmerzen oder visuellen Einschränkung und auch der Dauer der Schlauchintubation.

Emmerich et al. stellten 2009 fest, dass durch die Einführung der minimal-invasiven Techniken der Anteil der durchgeführten Dakryozystorhinostomien von 30 % auf knapp 10 % in den Zentren Hagen und Darmstadt gesunken ist [2].

Zwar ist die Anzahl der betrachteten Eingriffe unserer Klinik deutlich geringer, aber in dieser Analyse lag der Anteil der durchgeführten Dakryozystorhinostomien bei 65,7 % (111 von 169).

Dies kann evtl. durch die Selektion unseres Patientengutes erklärt werden. Zudem gibt es im näheren Umkreis diverse Einrichtungen, welche ebenfalls eine operative Versorgung anbieten.

In einer Studie von Heichel et al. wurden 387 Verläufe nach DCR aus den Jahren 2000 bis 2011 ausgewertet. Insgesamt zeigten sich 74,2 % sehr zufrieden mit dem Ergebnis und 20,2 % zufrieden [3]. Somit konnte die Arbeitsgruppe einen Langzeiterfolg von 94,4 % dokumentieren.

In unserer Patientengruppe, die eine DCR erhielt, zeigten sich 64,86 % der Patienten sehr zufrieden und 13,51 % zufrieden. Dies entspricht gemäß der Kalkulation von Heichel et al. einem Langzeiterfolg von 78,37 %.

In der Literatur werden für die DCR Erfolgsraten von 80–99 % angegeben [4]. Die Quote unseres Patientenkollektivs erreicht diesen Wert nahezu. Jedoch muss man hier deutlich anmerken, dass es sich bei unseren Daten um eine subjektive Einschätzung der Patienten handelt und es keine objektive Korrelation bzw. Nachuntersuchung zum Zeitpunkt der Datenerhebung gibt. Gegebenenfalls spielen auch gewisse geografische Unterschiede hierbei eine Rolle.

## Fazit für die Praxis

Zu konstatieren ist abschließend, dass die DCR trotz Hautschnitt und Knochenstanze mindestens als gleichwertig hinsichtlich der Patientenzufriedenheit anzusehen ist.
